# Supplementary material for: A chromosomal mutation is superior to a plasmid-encoded mutation for plasmid fitness cost compensation
Source: PLoS Biol. 2024 Dec 2;22(12):e3002926. doi: 10.1371/journal.pbio.3002926 (PMC11637435; doi:10.1371/journal.pbio.3002926)
Supplement: S1 File — Fig A. Example dynamics from different regions of parameter space shown in Fig 1. Numerical simulations of a continuous flow model using the following parameters: α = 0.5 h-1, uncompensated plasmid-bearer relative fitness = 0.82, compensated plasmid relative fitness = 0.95, K = 5.7 × 109 ml-1, μ = 0.04125. The base conjugation rate γ was set above the “domination” threshold γdom=μ(α−β)/(β−μ) at 9.9 × 10−12 ml.cells-1h-1 and was reduced below γdom or the “invasion” threshold γinv=μ(α−β)/(α−μ) for compensated and uncompensated plasmids-bearers to explore parameter space. The compensated plasmid conjugation rate γC = γQ was set at γ2 for cases where γC = γQ>γdom, at γ10.5 where γinv < γQ = γC<γdom, and at γ12 for cases where γQ = γC<γinv. The uncompensated plasmid conjugation γP rate was set at γ3.1 for γinv<γP<γdom and at γ3.5 where γP<γinv. Details on parameterisation are provided in Table A in S1 File. (A) Examples across parameter space for chrCM with initial density at 0.01 K, initial plasmid carriage at frequency of 50%, with 50% of these with CM. Coloured bars below each subpanel header indicate the corresponding regions of parameter space highlighted in Fig 1. (B) As (A) but for plaCM. (C) Different initial conditions for γinv<γP<γdom,γdom<γC (the pink region of Fig 1A). In both cases, plasmid-bearers start at 50% of the total, but in the left hand-panel the CM is at 50% of this population, whereas on the right it is 0.01%. The boundary for the initial conditions, solved analytically, is provided in Supplementary Text B in S1 File, and the data underlying this figure can be found in https://dx.doi.org/10.5281/zenodo.13963497. Fig B. Approximate Extended Simonsen conjugation rates for pQBR57 and pQBR57::plaCM. The dotted line indicates the previously measured conjugation rate for wild-type pQBR57 [12]. The data underlying this figure can be found in https://dx.doi.org/10.5281/zenodo.13963497. Fig C. Individual replicates for the summarized data presented in Fig [file pbio.3002926.s001.pdf]

Supplementary Information for:

A chromosomal mutation is superior to a plasmid-encoded mutation for plasmid fitness cost compensation

Rosanna C.T. Wright[1] 0000-0002-8095-8256, A. Jamie Wood[2,3] 0000-0002-6119-852X, Michael J. Bottery[4] 0000-0001-5790-1756, Katie J. Muddiman[1] 0000-0002-0396-6893, Steve Paterson[5] 0000-0002-1307-2981, Ellie Harrison[6] 0000-0002-2050-4631, Michael A. Brockhurst[1] 0000-0003-0362-820X, James P.J. Hall[5]* 0000-0002-4896-4592

1. Division of Evolution, Infection and Genomic Sciences, University of Manchester, Manchester, United Kingdom
2. Department of Biology, University of York, York, United Kingdom
3. Department of Mathematics, University of York, York, United Kingdom
4. Division of Infection, Immunity & Respiratory Medicine, University of Manchester, Manchester, United Kingdom
5. Department of Evolution, Ecology and Behaviour, Institute of Infection, Veterinary and Ecological Sciences, University of Liverpool, Liverpool, United Kingdom
6. School of Biosciences, University of Sheffield, Sheffield, United Kingdom

*Correspondence to j.p.j.hall@liverpool.ac.uk

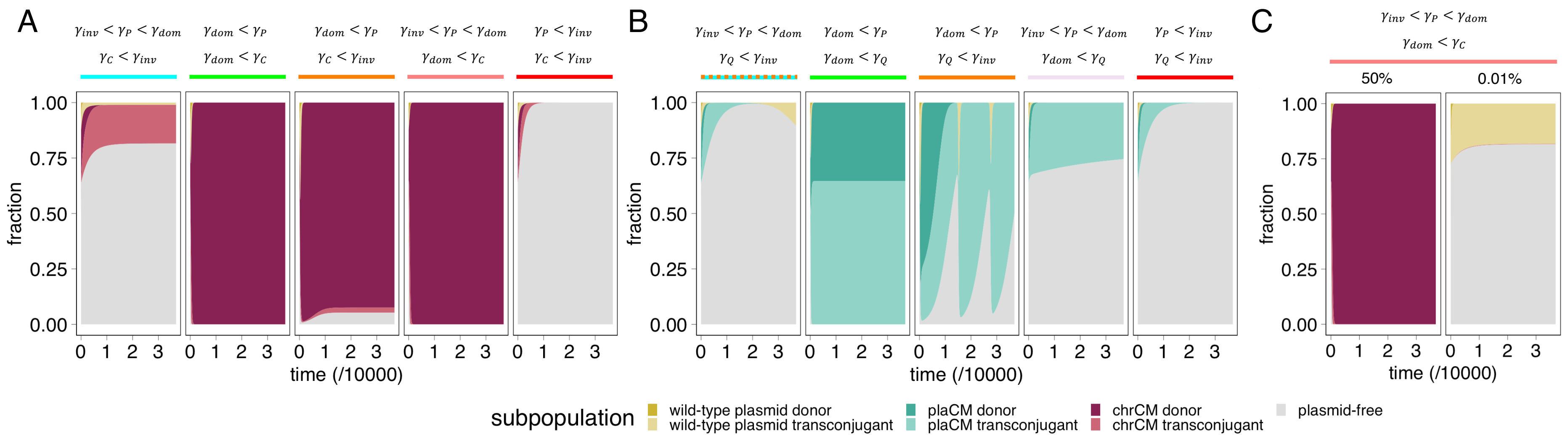

Figure A. Example dynamics from different regions of parameter space shown in Fig. 1. Numerical simulations of a continuous-flow model using the following parameters: α = 0.5 h^{-1}, uncompensated plasmid-bearer relative fitness = 0.82, compensated plasmid relative fitness = 0.95, K = 5.7 x 10^{9} ml^{-1}, μ = 0.04125. The base conjugation rate γ was set above the 'domination' threshold $\gamma_{dom} = \mu(\alpha - \beta)/(\beta - \mu)$ at 9.9 x 10^{-12} ml.cells^{-1}h^{-1} and was reduced below γ_{dom} or the 'invasion' threshold $\gamma_{inv} = \mu(\alpha - \beta)/(\alpha - \mu)$ for compensated and uncompensated plasmids-bearers to explore parameter space. The compensated plasmid conjugation rate $\gamma_C = \gamma_Q$ was set at $\frac{\gamma}{2}$ for cases where $\gamma_C = \gamma_Q > \gamma_{dom}$, at $\frac{\gamma}{10.5}$ where $\gamma_{inv} < \gamma_Q = \gamma_C < \gamma_{dom}$, and at $\frac{\gamma}{12}$ for cases where $\gamma_Q = \gamma_C < \gamma_{inv}$. The uncompensated plasmid conjugation γ_P rate was set at $\frac{\gamma}{3.1}$ for $\gamma_{inv} < \gamma_P < \gamma_{dom}$ and at $\frac{\gamma}{3.5}$ where $\gamma_P < \gamma_{inv}$. Details on parameterisation are provided in Table A in S1 File. (A) Examples across parameter space for chrCM with initial density at 0.01K, initial plasmid carriage at frequency of 50%, with 50% of these with CM. Coloured bars below each subpanel header indicate the corresponding regions of parameter space highlighted in Fig. 1. (B) As (A) but for plaCM. (C) Different initial conditions for $\gamma_{inv} < \gamma_P < \gamma_{dom}, \gamma_{dom} < \gamma_C$ (the pink region of Fig. 1A). In both cases plasmid-bearers start at 50% of the total, but in the left hand-panel the CM is at 50% of this population, whereas on the right it is 0.01%. The boundary for the initial conditions, solved analytically, is provided in the Appendix, and the data underlying this Figure can be found in https://dx.doi.org/10.5281/zenodo.13963497

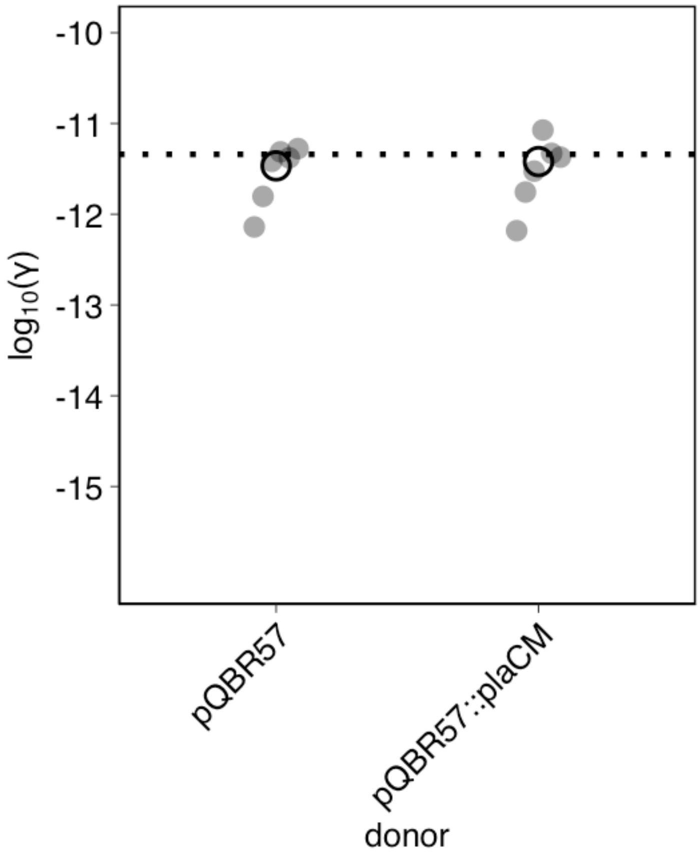

Figure B. Approximate Extended Simonsen conjugation rates for pQBR57 and pQBR57::plaCM. The dotted line indicates the previously-measured conjugation rate for wild-type pQBR57 [1]. The data underlying this Figure can be found in https://dx.doi.org/10.5281/zenodo.13963497

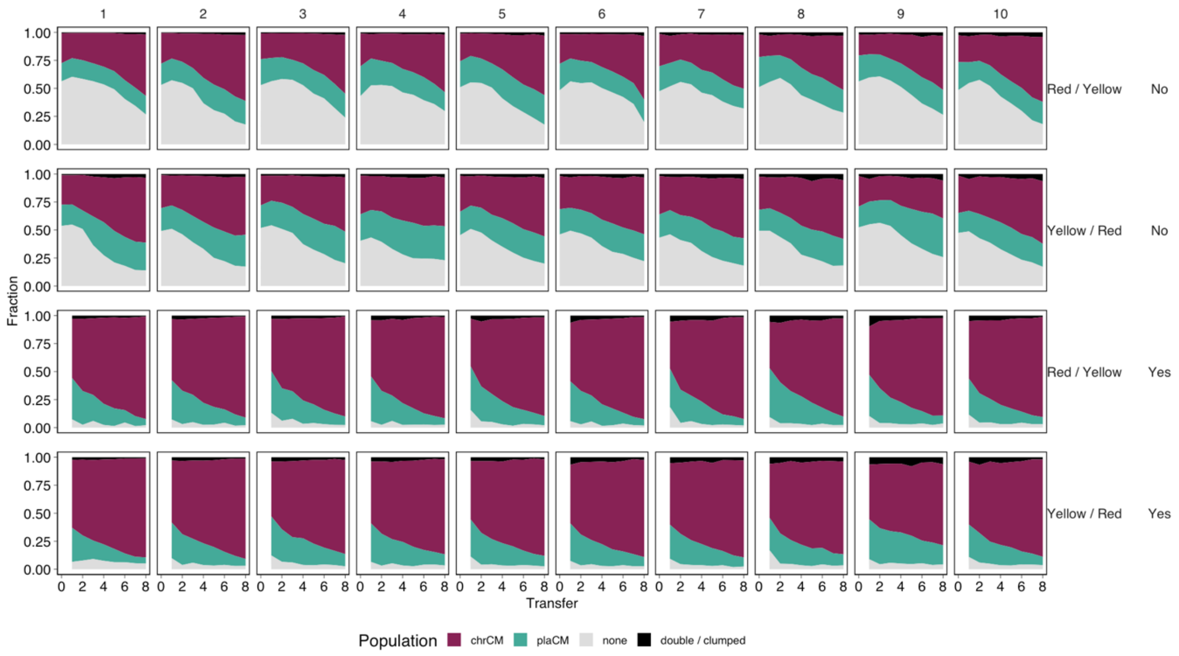

Figure C. Individual replicates for the summarized data presented in Fig. 3. Red/Yellow and Yellow/Red refer to the orientation of the fluorescent markers (chrCM / plaCM), and Yes / No refers to mercury selection. The data underlying this Figure can be found in https://dx.doi.org/10.5281/zenodo.13963497

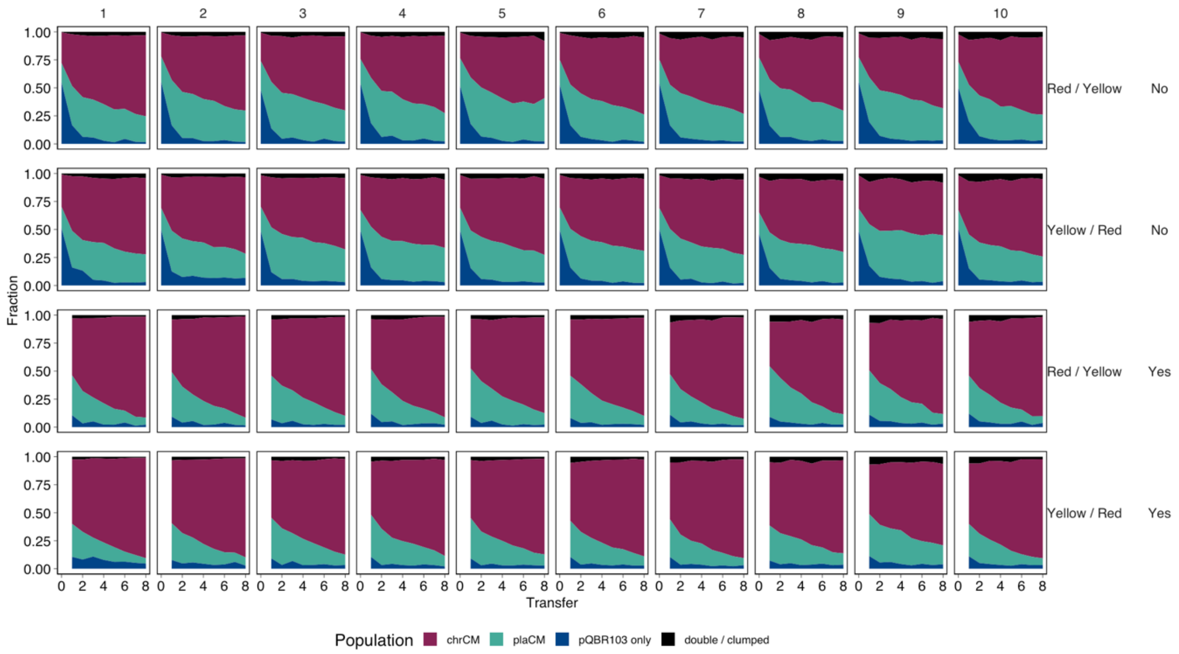

Figure D. Individual replicates for the summarized data presented in Fig. 4. Red/Yellow and Yellow/Red refer to the orientation of the fluorescent markers (chrCM / plaCM), and Yes / No refers to mercury selection. The data underlying this Figure can be found in https://dx.doi.org/10.5281/zenodo.13963497

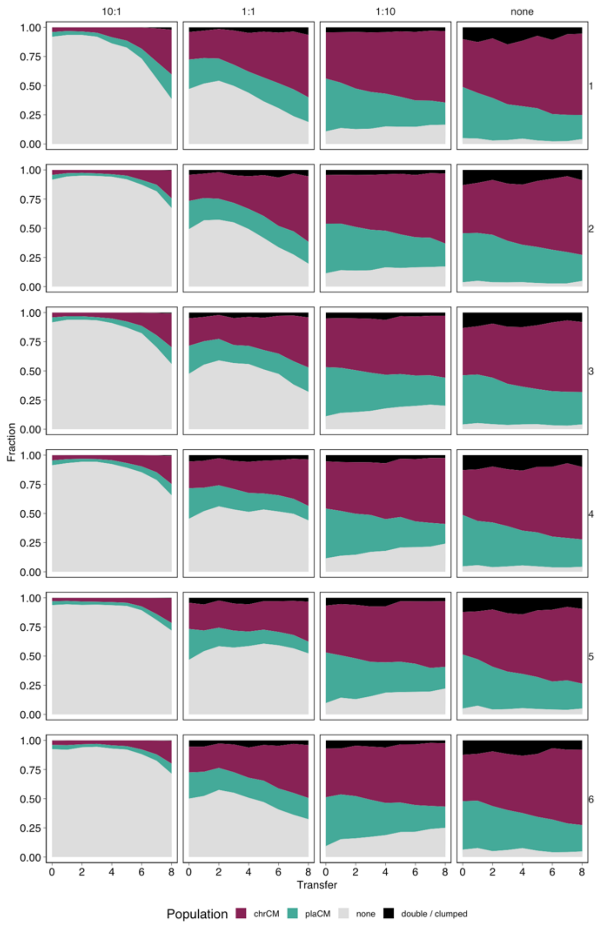

Figure E. Individual replicates for the summarised data presented in Fig. 5. The data underlying this Figure can be found in https://dx.doi.org/10.5281/zenodo.13963497

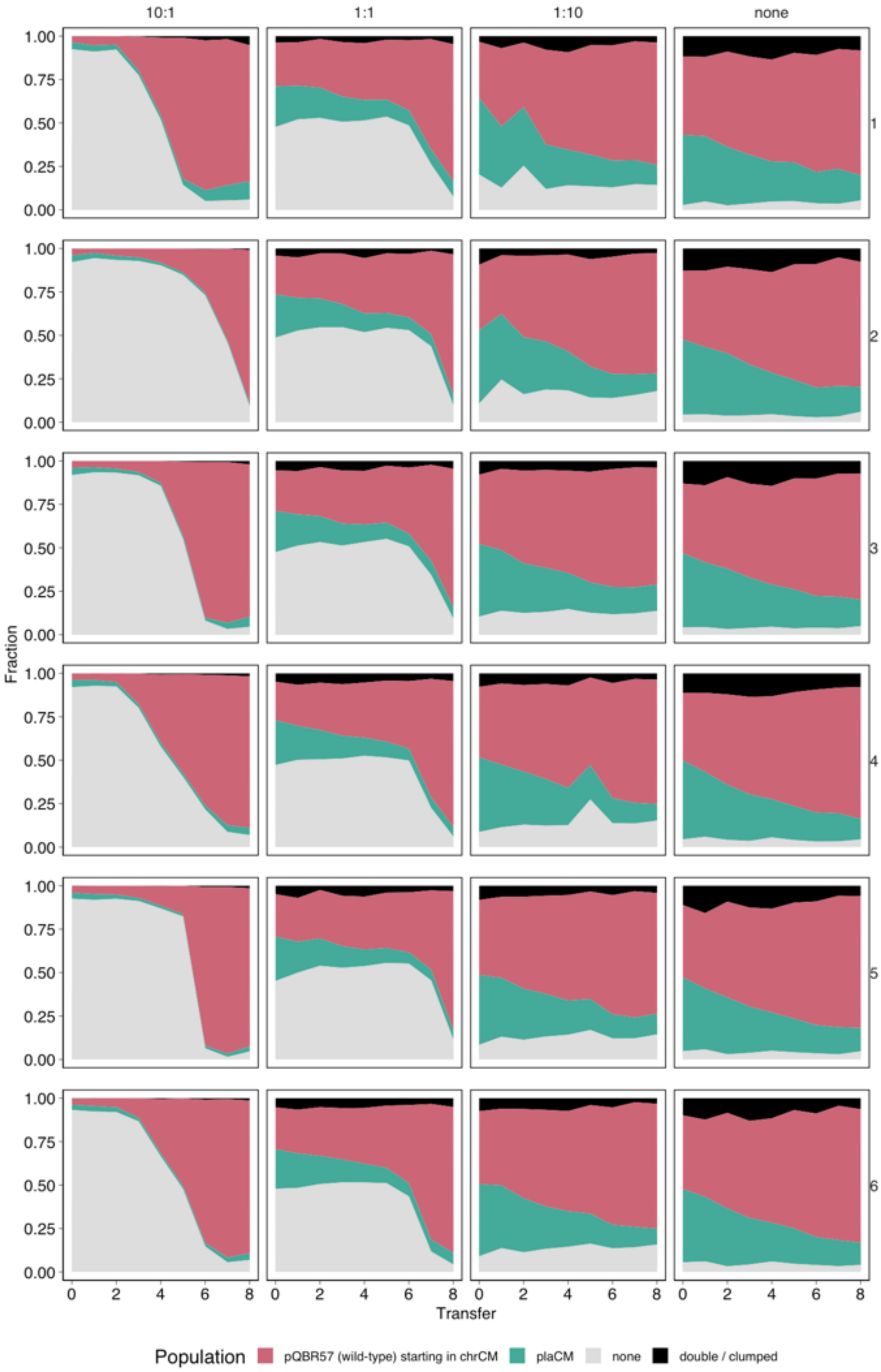

Figure F. Individual replicates for the summarised data presented in Fig. 6. The data underlying this Figure can be found in https://dx.doi.org/10.5281/zenodo.13963497

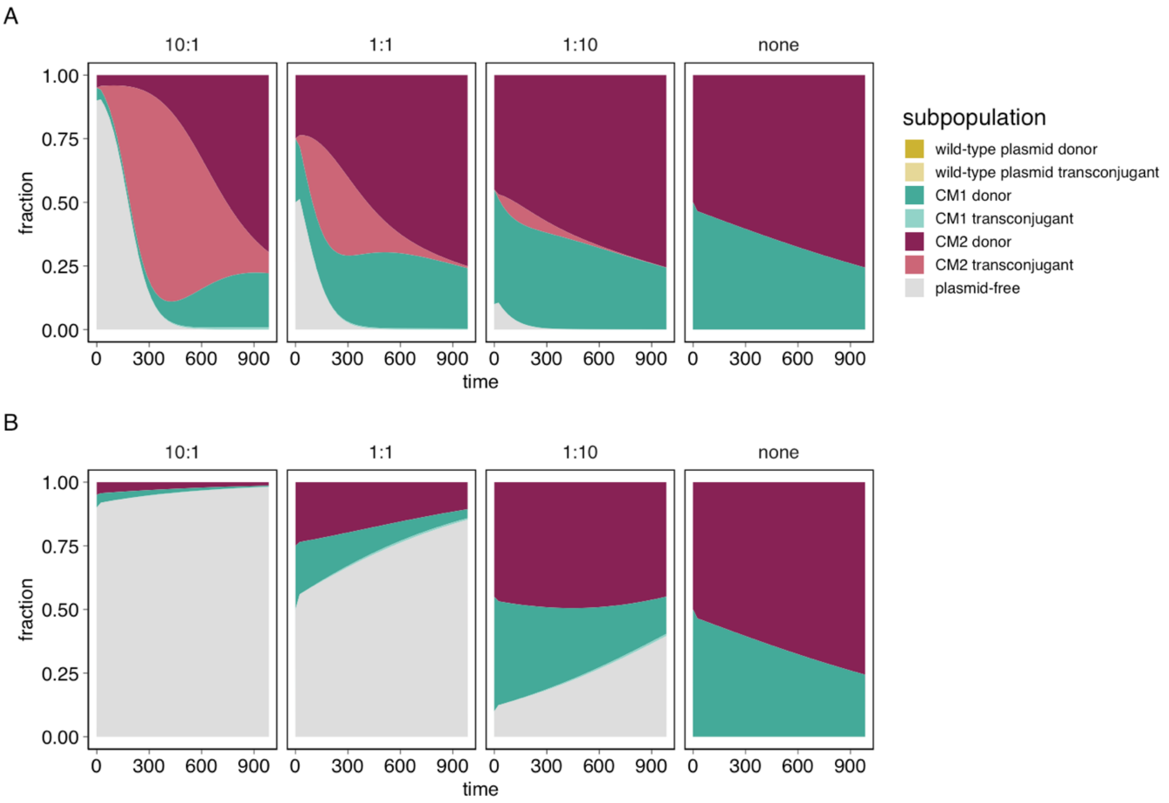

Figure G. (A) ODE-based model simulations resemble plaCM versus chrCM experimental results in main text Figs 3, 5 and 6. Numerical simulations of a continuous flow model based on the following parameters: α = 0.54 h^{-1}, SBW25::chrCM(pQBR57) relative fitness = 0.97, SBW25(pQBR57::plaCM) relative fitness = 0.95, K = 5.7 x 10^{9} ml^{-1}, chrCM conjugation rate γ_C = 4.6 x 10^{-12} ml.cells^{-1}h^{-1}, plaCM conjugation rate $\gamma_Q = \frac{\gamma_C}{100}$ (see main text and Fig. I in S1 File for discussion). Details on parameterisation are provided in Table A in S1 File. (B) Plasmid weaponisation can drive outcomes. As panel A, except chrCM conjugation rate γ_C was set to zero. The qualitatively different outcomes between panels A and B demonstrate the role that costly plasmid transmission from compensated strains can play. An interactive version of this figure is provided by following the link at www.jpjhall.net/plasmid-dynamics-model. The code underlying this Figure can be found in https://dx.doi.org/10.5281/zenodo.13963497

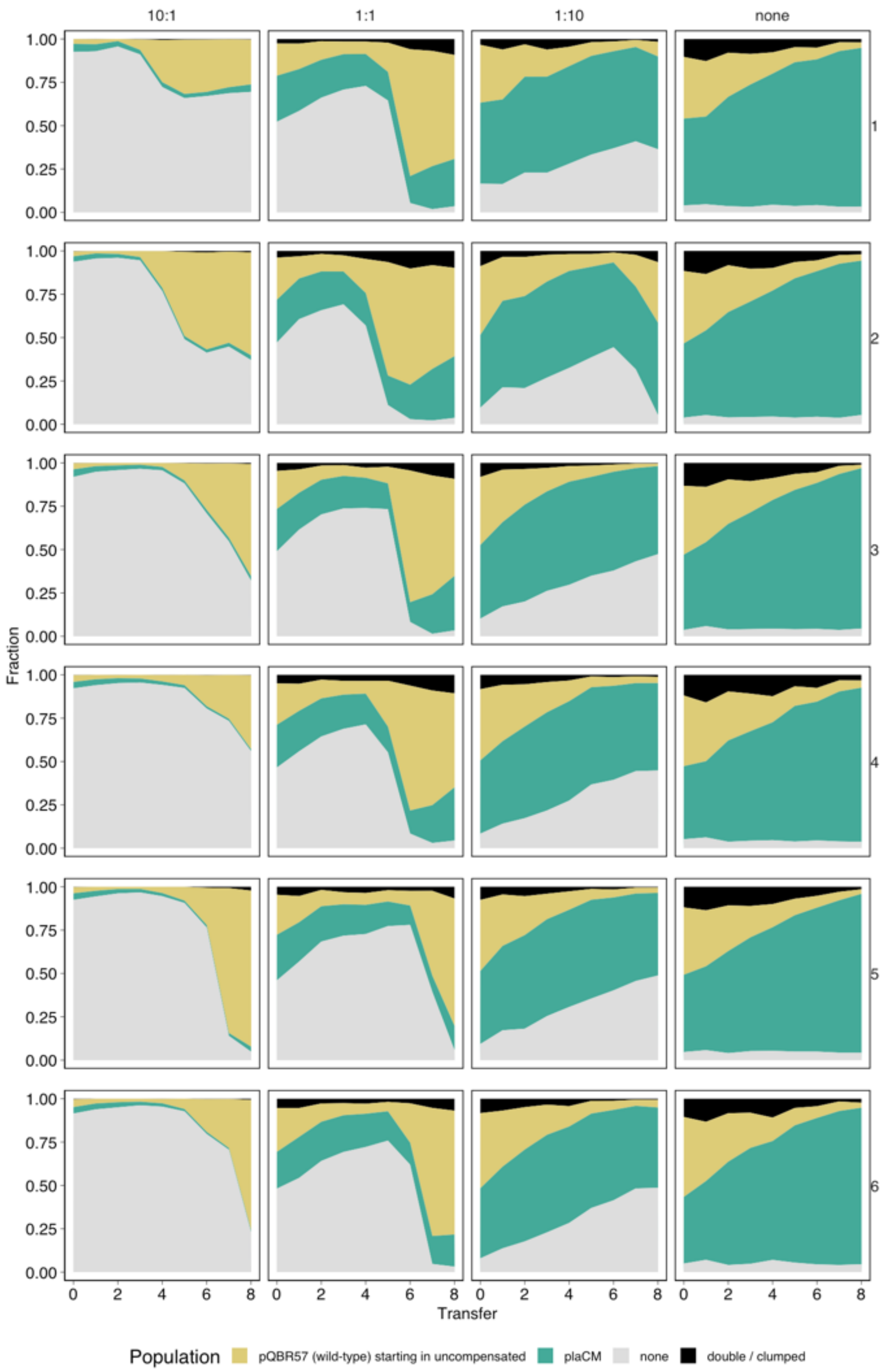

Figure H. Individual replicates for the summarised data presented in Fig. 7. The data underlying this Figure can be found in https://dx.doi.org/10.5281/zenodo.13963497

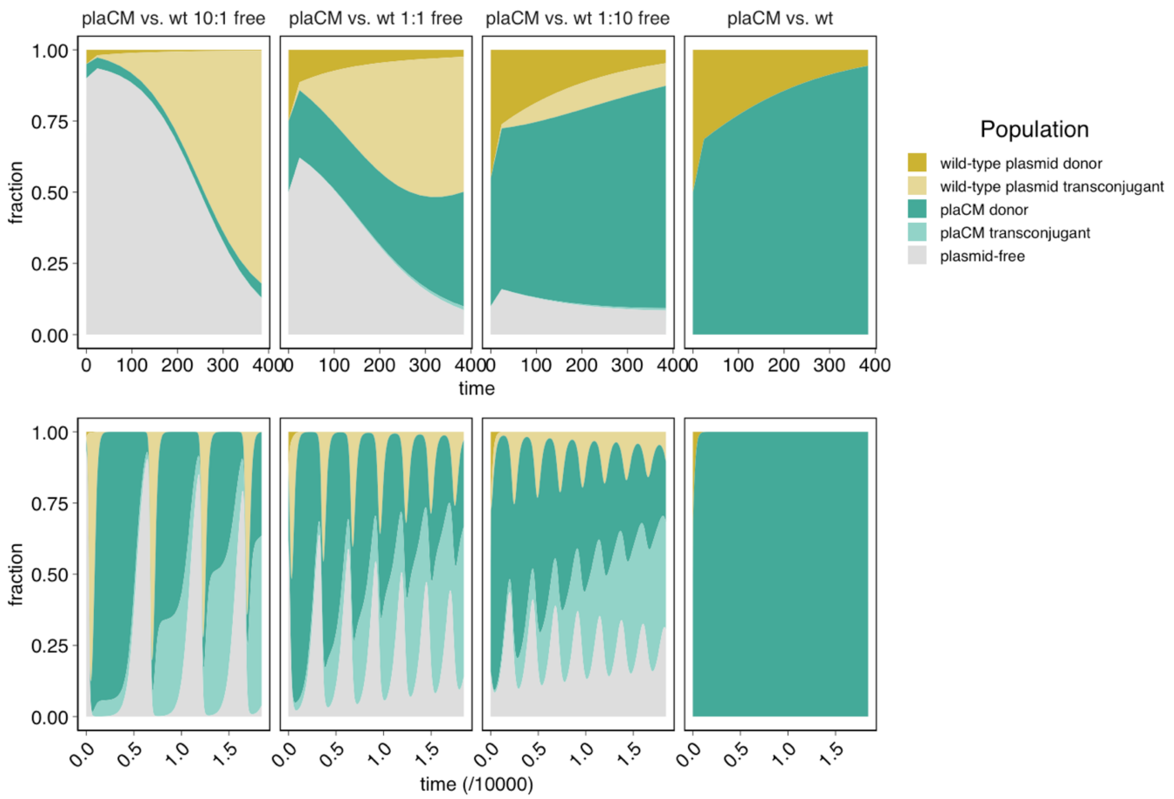

Figure I. ODE-based model simulations resemble experimental results for low $\frac{\gamma_Q}{\gamma_P}$ ratios.

Numerical simulations of a continuous flow model based on the following parameters: α = 0.54 h^{-1}, SBW25(pQBR57) relative fitness = 0.82, SBW25(pQBR57::plaCM) relative fitness = 0.95, K = 5.7 x 10^{9} ml^{-1}, uncompensated conjugation rate γ_P = 4.6 x 10^{-12} ml.cells^{-1}h^{-1}, plaCM conjugation rate $\gamma_Q = \frac{\gamma_P}{100}$. Top panels indicate short term dynamics, bottom panels longer-term dynamics. Details on parameterisation are provided in Table A in S1 File. An interactive version of this figure is provided by following the link at www.jpjhall.net/plasmid-dynamics-model. The code underlying this Figure can be found in https://dx.doi.org/10.5281/zenodo.13963497

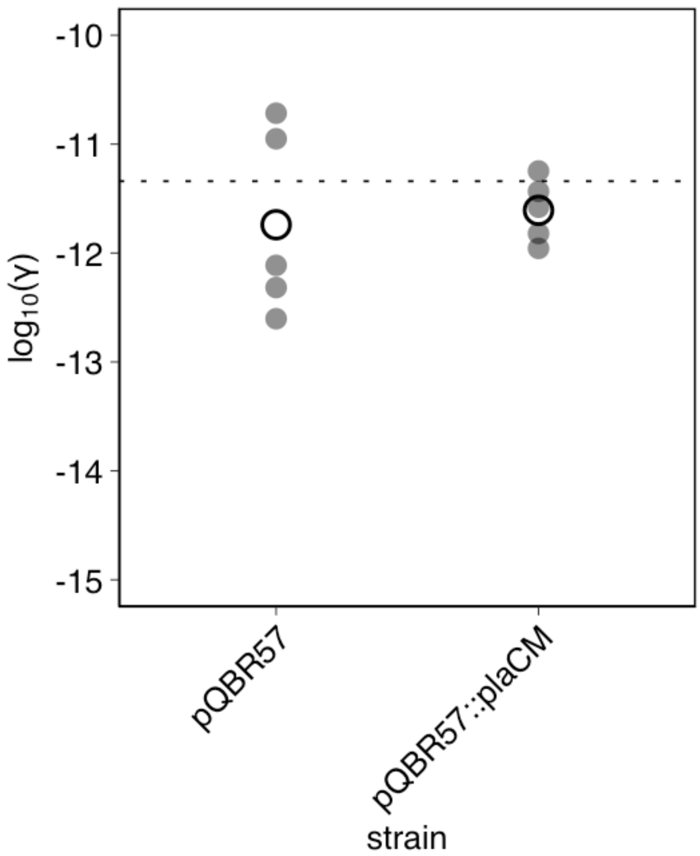

Figure J. An excess of recipients does not favour wild-type pQBR57. Conjugation experiments were established with a 100x excess of recipients and run for 18 hours. The dotted line indicates the previously-measured conjugation rate for wild-type pQBR57 [1]. The data underlying this Figure can be found in https://dx.doi.org/10.5281/zenodo.13963497

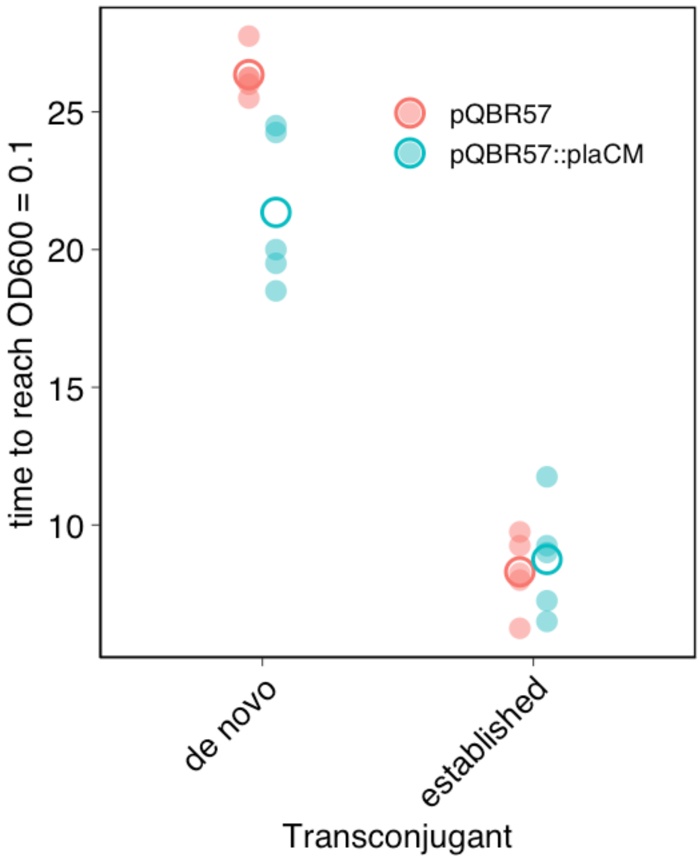

Figure K. *De novo* plaCM transconjugants do not suffer increased lag time. Growth curves were conducted with fresh transconjugants under selection. PlaCM had a significantly reduced lag time compared with wild-type pQBR57 ($t_{4.71}$ = 3.81, p = 0.014), but only in the *de novo* transconjugants. Time is in hours. The data underlying this Figure can be found in https://dx.doi.org/10.5281/zenodo.13963497

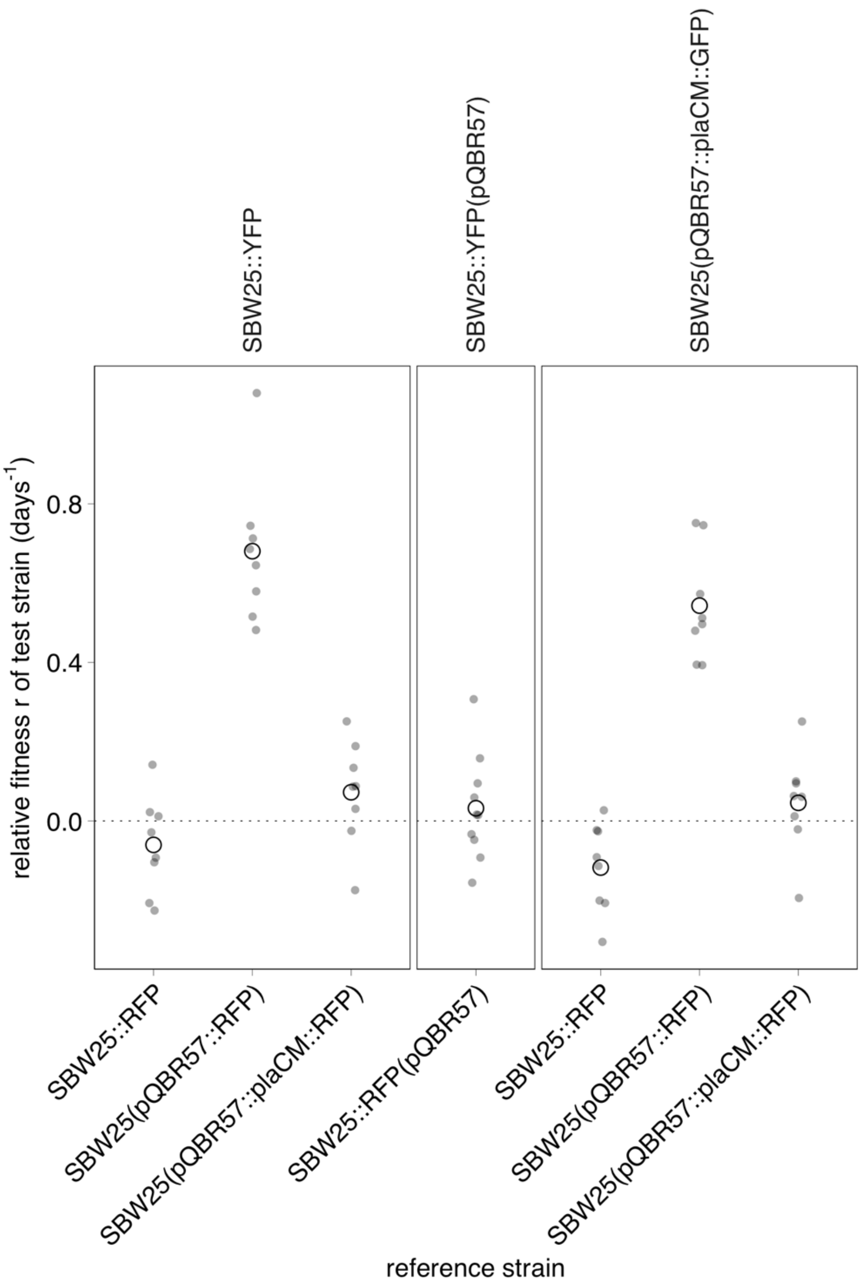

Figure L. Control experiments testing fluorescently-labelled strains produced results consistent with expectations. Unfilled circles indicate the mean across 10 (where test = SBW25::YFP(pQBR57)) or 8 replicates. Competitions between differently labelled isogenic-labelled strains all produced non-significant results ($p > 0.2$) and small coefficients (< 0.061) by t-test when comparing with $r = 0$, and uncompensated plasmid fitness costs were recapitulated in competition against wild-type (SBW25::YFP) and plaCM ($p < 0.00002$ in both cases, coefficients 0.68, and 0.54 respectively). A small (coefficient 0.12), marginally significant cost of plaCM ($p = 0.021$) was detected in competition with plasmid-free (SBW25::RFP), consistent with other observations that plaCM amelioration may be less complete than alternative CMs. The data underlying this Figure can be found in https://dx.doi.org/10.5281/zenodo.13963497

Table A. Experimentally measured parameters used to inform numerical simulations

Parameter*	Units	Value	Standard Deviation	Notes and source data
α	h^{-1}	0.541	0.0553	Calculated using gcplyr [2] from growth curve data collected by subculturing overnight cultures 1:1000 into 150 µl fresh KB media and measuring optical density at 600 nm using a Tecan Nano plate reader every 15 minutes for 24 hours. Mean of 12 replicate cultures.
SBW25(pQBR57) relative fitness	dimensionless	0.818	0.0429	[1]. Multiply by α for β_P.
SBW25::chrCM(pQBR57) relative fitness	dimensionless	0.969	0.0699	[1]. Multiply by α for β_C.
SBW25(pQBR57::plaCM) relative fitness	dimensionless	0.945	0.0413	[1]. Multiply by α for β_Q.
K	ml^{-1}	5.72e9	1.59e9	[1]
pQBR57 conjugation rate	ml/cells/h	4.57e-12	1.014e-12	[1]. Multiply by K to give γ in the (scaled) analytic model.
μ	h^{-1}	0.04125	0	Calculated as an average considering a 1:100 dilution/24h.

Supplementary Text A

The following equations describe the numerical simulation model presented by following the link at www.jpjhall.net/plasmid-dynamics-model and Figs. A, G and I in S1 File. This model has been generalised to flexibly describe different types of compensatory mutation (CM). Here, Z_f describes the wild-type plasmid-free population, X_0 describes the wild-type population with the wild-type plasmid, X_1 describes a plasmid-containing population with CM type 1, and X_2 describes a plasmid-containing population with CM type 2. X_{0t}, X_{1t}, and X_{2t} describe transconjugants formed by transfer into Z_f from X_0, X_1, and X_2 respectively. Each population has its own growth rate, conjugation rate, and susceptibility to positive selection, and by adjusting these parameters, the features of plaCM and chrCM can be described. Specifically, to describe a plaCM, the growth rate of the transconjugant is set to be the same as that of the donor (e.g. $\alpha_{X_{1t}} = \alpha_{X_1}$), whereas to describe a chrCM, the growth rate of the transconjugant is set to be the same as that of X_0, the wild-type population with the wild-type plasmid (e.g. $\alpha_{X_{2t}} = \alpha_{X_0}$).

$$\sigma = 1 - \frac{(Z_f + X_0 + X_1 + X_2 + X_{0t} + X_{1t} + X_{2t})}{K}$$

$$\frac{dZ_f}{dt} = \alpha_{Z_f}\sigma Z_f - \mu Z_f - \gamma_{X_0}X_0Z_f - \gamma_{X_1}X_1Z_f - \gamma_{X_2}X_2Z_f - \gamma_{X_{0t}}X_{0t}Z_f - \gamma_{X_{1t}}X_{1t}Z_f - \gamma_{X_{2t}}X_{2t}Z_f - \eta_{Z_f}Z_f$$

$$\frac{dX_0}{dt} = \alpha_{X_0}\sigma X_0 - \mu X_0 - \eta_{X_0}X_0$$

$$\frac{dX_1}{dt} = \alpha_{X_1}\sigma X_1 - \mu X_1 - \eta_{X_1}X_1$$

$$\frac{dX_2}{dt} = \alpha_{X_2}\sigma X_2 - \mu X_2 - \eta_{X_2}X_2$$

$$\frac{dX_{0t}}{dt} = \alpha_{X_{0t}}\sigma X_{0t} - \mu X_{0t} + \gamma_{X_0}X_0Z_f + \gamma_{X_{0t}}X_{0t}Z_f - \eta_{X_{0t}}X_{0t}$$

$$\frac{dX_{1t}}{dt} = \alpha_{X_{1t}}\sigma X_{1t} - \mu X_{1t} + \gamma_{X_1}X_1Z_f + \gamma_{X_{1t}}X_{1t}Z_f - \eta_{X_{1t}}X_{1t}$$

$$\frac{dX_{2t}}{dt} = \alpha_{X_{2t}}\sigma X_{2t} - \mu X_{2t} + \gamma_{X_2}X_2Z_f + \gamma_{X_{2t}}X_{2t}Z_f - \eta_{X_{2t}}X_{2t}$$

(1)

Simulations were run using deSolve and rootSolve [3,4]

1 Basic Model

The starting point is to first analyse the well known pair of dimensionless equations

$$\begin{aligned}\frac{df}{dt} =& \alpha f(1-f-p) - \mu f - \gamma p f\\ \frac{dp}{dt} =& \beta p(1-f-p) - \mu p + \gamma p f,\end{aligned} \tag{1}$$

which represent the growth of a plasmid free strain, f, and a plasmid containing strain, p in the absence of selection. The strains have growth rate α and β. There is a washout rate μ and a conjugation rate γ. We take $\alpha > \beta > \mu$. A Jacobian matrix an be calculated to determine stability and is

$$J(f,p) = \begin{bmatrix} \alpha(1-2f-p) - \mu - \gamma p & -\alpha f - \gamma f \\ -\beta p + \gamma f & \beta(1-f-2p) - \mu + \gamma f \end{bmatrix} \tag{2}$$

This system has 4 fixed points which are

- $(0,0)$, this fixed point – no bacteria – is always unstable due to our assumptions
- $(f^*, 0)$, this fixed point with $f^* = 1 - \dfrac{\mu}{\alpha}$ – no plasmid – is stable when $\gamma < \frac{\mu(\alpha-\beta)}{\alpha-\mu}$
- $(0, p^*)$, this fixed point with $p^* = 1 - \dfrac{\mu}{\beta}$– plasmid dominates – is stable when $\gamma > \frac{\mu(\alpha-\beta)}{\beta-\mu}$
- (f^*, p^*), this fixed point with $p^* = \frac{\alpha}{\gamma z} - \frac{\mu}{\gamma}$ and $f^* = \frac{\mu}{\gamma} - \frac{\beta}{\gamma z}$ (where we define $z = \alpha - \beta + \gamma$) – a mixed solution – is stable when $\frac{\mu(\alpha-\beta)}{\alpha-\mu} < \gamma < \frac{\mu(\alpha-\beta)}{\beta-\mu}$

This gives a clear explanation for the expected dynamics as γ varies: for γ small conjugation is insufficient for the plasmid containing strain to invade the faster growing plasmid free strain and is eliminated. For large γ the opposite occurs and the plasmid containing strain is able to infectiously invade and dominate the population, eliminating the plasmid free strain. For a range of intermediate values of γ given by the inequality above, a balance is achieved between the growth rate advantage of the plasmid frees and them achieving sufficient levels in the population that they become infected with plasmids leading to coexistence.

In the analysis that follows we define the following terms for algebraic convenience:

- $z_P = \frac{\alpha - \beta_P + \gamma_P}{\gamma_P}$ and corresponding ratios for Q and C with the appropriate subscripts
- $\Omega = \beta_Q \gamma_P - \beta_P \gamma_Q$

2 Chromosome compensation model

The first case we consider is if a strain emerges which is assumed to carry a mutation that compensates the cost of the plasmid with this mutation occurring on the chromosome. The new strain has a population fraction c, is assumed to be baring a plasmid and its properties are subscripted C, as opposed to P for the original strain. In addition this strain has a differing plasmid conjugation rate γ_C as compared to γ_P. The full system has dynamics

$$\begin{aligned}\frac{df}{dt} =& \alpha f(1-f-p-c) - \mu f - \gamma_P p f - \gamma_C c f \\ \frac{dp}{dt} =& \beta_P p(1-f-p-c) - \mu p + \gamma_P p f + \gamma_C c f \\ \frac{dc}{dt} =& \beta_C c(1-f-p-c) - \mu c. \end{aligned} \tag{3}$$

To match our assumptions we have chosen $\beta_P < \beta_C < \alpha$ (WLOG mathematically). Taking these inequalities forward we find the following Jacobian matrix and fixed points:

$$J(f,p,c) = \begin{bmatrix} \alpha(\Delta - f) - \mu - \gamma_P p - \gamma_C c & -\alpha f - \gamma_P f & -\alpha f - \gamma_C f \\ -\beta_P p + \gamma_P p + \gamma_C c & \beta_P(\Delta - p) - \mu + \gamma_P f & -\beta_P p + \gamma_C f \\ -\beta_C c & -\beta_C c & \beta_C(\Delta - c) - \mu \end{bmatrix} \tag{4}$$

with $\Delta = 1 - f - p - c$.

- $(0,0,0)$, this fixed point – no bacteria – is always unstable due to our assumptions
- $(f^*,0,0)$, this fixed point, $f^* = 1 - \dfrac{\mu}{\alpha}$ – no plasmid – is stable when $\gamma_P < \frac{\mu(\alpha - \beta_P)}{\alpha - \mu}$.
- $(0,p^*,0)$, this fixed point, $p^* = 1 - \dfrac{\mu}{\beta_P}$ – original plasmid only – is always unstable
- $(f^*,p^*,0)$, a fixed point, $f^* = \frac{\alpha}{\gamma_Q z_Q} - \frac{\mu}{\gamma_Q}$, $p^* = \frac{\mu}{\gamma_Q} - \frac{\beta_Q}{\gamma_Q z_Q}$ – a mixed solution between frees and original plasmids.
- $(0,0,c^*)$, this fixed point, $c^* = 1 - \dfrac{\mu}{\beta_C}$ – new plasmid dominates – is stable when $\gamma_C > \frac{\mu(\alpha - \beta_C)}{\beta_C - \mu}$
- (f^*,p^*,c^*), a fixed point – a full mixed coexistence solution between all three strains.

This fully mixed fixed point is given by $f^* = \frac{(\beta_C - \beta_P)\gamma_C}{\beta_C \Sigma}(\beta_C - \mu z_C)$, $p^* = \frac{(\alpha - \beta_C)\gamma_C}{\beta_C \Sigma}(\beta_C - \mu z_C)$ and $c^* = \frac{(\alpha - \beta_C)\gamma_C}{\beta_C \Sigma}(\mu z_P - \beta_C)$ and we define $\Sigma = \Omega - \alpha(\gamma_P - \gamma_C)$ for convenience and Ω and $z_{<.>}$ as before. This fixed point has two differing roles. When γ_P is large and γ_C small this is the stable fixed point which may or may not be oscillatory in character. A more applicable role is it acts as a boundary (it is a saddle in this situation, and notated (f_s, p_s, c_s)) between two stable fixed points in the region marked "3 fixed points". This creates the possibility for dependence on initial conditions in this region and the inability of a chromosomal mutation to invade unless it does so with sufficient numbers.

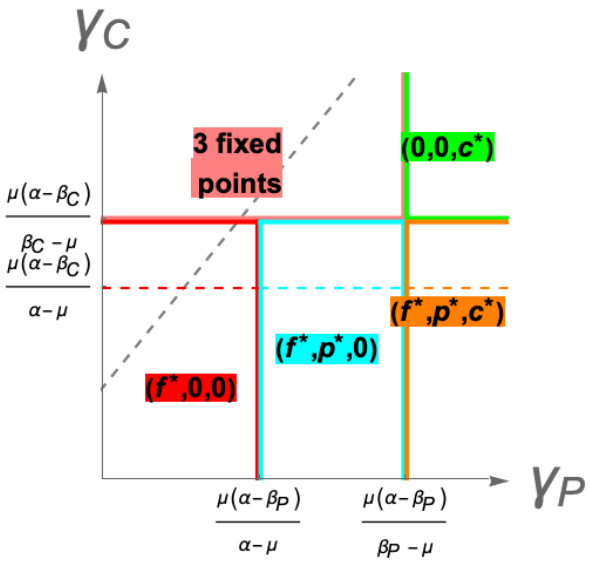

Figure 1: Phase diagram for chromosome compensation

3 Plasmid compensation model

The second variant we consider is if another strain emerges which is assumed to carry a mutation that compensates the cost of the plasmid but with mutation occurring on the plasmid. The new strain has a population fraction q and its properties are subscripted Q, as apposed to P for the original strain. The new plasmid has a differing conjugation rate γ_Q as compared to γ_P. The full system

has dynamics

$$\begin{aligned}\frac{df}{dt} =& \alpha f(1-f-p-q) - \mu f - \gamma_P pf - \gamma_Q qf \\ \frac{dp}{dt} =& \beta_P p(1-f-p-q) - \mu p + \gamma_P pf \\ \frac{dq}{dt} =& \beta_Q q(1-f-p-q) - \mu q + \gamma_Q qf, \end{aligned} \tag{5}$$

To match our assumptions we have chosen $\beta_P < \beta_Q \leq \alpha$ (WLOG mathematically). If the new conjugation rate $\gamma_Q \geq \gamma_P$ then the new strain is simply better than the old and will quickly displace it and we return to the basic model first described (with p exchanged with q). Therefore interesting dynamics are only possible for $\gamma_Q < \gamma_P$. Taking these inequalities forward we find the following Jacobian matrix and fixed points:

$$J(f,p,q) = \begin{bmatrix} \alpha(\Delta-f)-\mu-\gamma_P f-\gamma_Q q & -\alpha f-\gamma_P f & -\alpha f-\gamma_Q f \\ -\beta_P p+\gamma_P p & \beta_P(\Delta-p)-\mu+\gamma_P f & -\beta_P p \\ -\beta_Q c+\gamma_Q q & -\beta_Q q & \beta_Q(\Delta-q)-\mu+\gamma_Q f \end{bmatrix} \tag{6}$$

with $\Delta = 1-f-p-q$.

- $(0,0,0)$, this fixed point – no bacteria – is always unstable due to our assumptions
- $(f^*,0,0)$, this fixed point, $f^* = 1-\frac{\mu}{\alpha}$ – no plasmid – is stable when $\gamma_P < \frac{\mu(\alpha-\beta_P)}{\alpha-\mu}$ and $\gamma_Q < \frac{\mu(\alpha-\beta_Q)}{\alpha-\mu}$
- $(0,p^*,0)$, this fixed point, $p^* = 1-\frac{\mu}{\beta_P}$ – original plasmid dominates – is never stable because of our assumption that $\beta_Q > \beta_P$.
- $(0,0,q^*)$, this fixed point, $q^* = 1-\frac{\mu}{\beta_Q}$ – new plasmid dominates – is stable when $\gamma_Q > \frac{\mu(\alpha-\beta_Q)}{\beta_Q-\mu}$
- $(0,p^*,q^*)$, this fixed point – a mixed plasmid solution – never exists due to our assumption that $\beta_P \neq \beta_Q$

In addition there are three further fixed points for which analytic progress is more challenging

- $(f^*,p^*,0)$, a fixed point – a mixed solution between frees and original plasmids.
- $(f^*,0,q^*)$, a fixed point – a mixed solution between frees and new plasmids.

- (f^*, p^*, q^*), a fixed point – a fully mixed solution.

The first two fixed points are mathematically symmetric to each other. In all cases interchanging $P \leftrightarrow Q$ will give the conditions for $(f^*, p^*, 0)$. We consider $(f^*, 0, q^*)$ WLOG, where $f^* = \frac{\mu}{\gamma_Q} - \frac{\beta_Q}{\gamma_Q z_Q}$ and $q^* = \frac{\alpha}{\gamma_Q z_Q} - \frac{\mu}{\gamma_Q}$ and we use the definition $z_Q = \frac{\alpha - \beta_Q + \gamma_Q}{\gamma_Q}$ for convenience. This fixed point is physical in the band $\frac{\mu(\alpha-\beta_Q)}{\alpha-\mu} < \gamma_Q < \frac{\mu(\alpha-\beta_Q)}{\beta_Q-\mu}$. Computing the eigenvalues of the Jacobian matrix leads to factorisation of the characteristic polynomial to the eigenvalue

$$\lambda_Q = \mu\frac{(\gamma_P - \gamma_Q)}{\gamma_Q} - \frac{\Omega}{\gamma_Q z_Q} \quad \text{where} \quad \Omega = \beta_Q\gamma_P - \beta_P\gamma_Q \tag{7}$$

and a quadratic. Solving the quadratic is not revealing but the Routh-Horwitz conditions lead to conditions corresponding to the existence conditions (see included Mathematica worksheet). Therefore this fixed point (and the corresponding one) are stable when they exists and when $\lambda_Q < 0$.

The fully mixed fixed point (f^*, p^*, q^*), where $f^* = \frac{\mu(\beta_Q-\beta_P)}{\Omega}$, $p^* = \frac{\mu\gamma_Q z_Q}{\Omega} - \frac{\gamma_Q}{\gamma_P-\gamma_Q}$ and $q^* = \frac{\gamma_P}{\gamma_P-\gamma_Q} - \frac{\mu\gamma_P z_P}{\Omega}$ is not analytically tractable but the existence conditions correspond to when the eigenvalues are negative implying, by continuity considerations, that the fully mixed point is also stable when it exists. The nature of the fixed point is inaccessible through explicit analytic computation but it can either be a stable node or a stable oscillatory node according to the sign of the discriminant. This can be demonstrated by examining the characteristic polynomial of the Jacobian matrix evaluated at the fixed point using computational algebra, a mathematica notebook is provided for this purpose. The Routh-Hurwitz conditions apply which means the root must always be negative. This means that no unstable fixed can emerge via a Hopf bifurcation so no limit cycle is possible.

A conventional explanation would assert that due to competitive exclusion the fully mixed solution should never be stable. An adaptive dynamics interpretation would only suggest that the linear invasion of the new plasmid would occur when introduced, and then implicitly that the invader should sweep to fixation. Instead this full analysis, accompanied by numerical simulation results, indicates the existence of a complete coexistence regime for biologically relevant parameters.

4 Selection

Selection can easily be imposed by adding a higher turnover term for any sensitive species. At its simplest, if we assume that the mechanism of resistance is identical, and is not affected by any subsequent compensation, this can be included as a linear loss term, proportional to η on the plasmid free species.

For the basic model this gives the following equations:

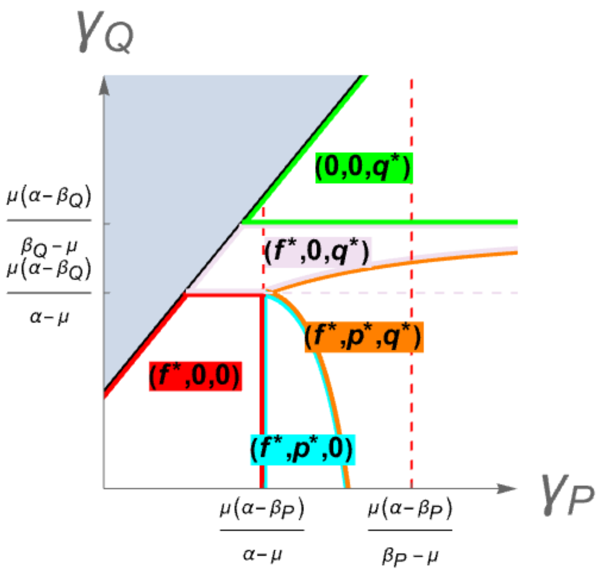

Figure 2: Phase diagram for plasmid compensation

$$\begin{aligned}\frac{df}{dt} =& \alpha f(1-f-p) - \mu f - \gamma p f - \eta f \\ \frac{dp}{dt} =& \beta p(1-f-p) - \mu p + \gamma p f,\end{aligned} \tag{8}$$

which shifts the free only fixed point to $1-\frac{\mu+\eta}{\alpha}$ and the mixed fixed point to $f_s^* = f^* - \eta\left(\frac{\beta}{z\gamma^2}\right)$,$p_s^* = p^* + \eta\left(\frac{\beta-\gamma}{z\gamma^2}\right)$, shifting the solution toward plasmid dominance, as expected.

For the plasmid compensation system the equations become

$$\begin{aligned}\frac{df}{dt} =& \alpha f(1-f-p-q) - \mu f - \gamma_P p f - \gamma_Q q f - \eta f \\ \frac{dp}{dt} =& \beta_P p(1-f-p-q) - \mu p + \gamma_P p f \\ \frac{dq}{dt} =& \beta_Q q(1-f-p-q) - \mu q + \gamma_Q q f,\end{aligned} \tag{9}$$

and analogously for the chromosome compensation system. In both cases the analysis can be computed exactly but the results do not change our interpretation. For small values of η the findings are unchanged. A selection limit, η^* exists (equal to the maximum of two value determined by the differing plasmids) where if selection is greater than both of these values then plasmid free states cannot exist and therefore the fittest plasmid solution always wins, which will

be the compensated state, but it may take a long time due to relative absence of plasmid free target to facilitate spread. For the plasmid compensation

$$\eta_Q^* = \frac{z_Q \gamma_Q \mu - \beta_Q \gamma_Q}{\beta_Q} \tag{10}$$

whilst for the chromosome compensation

$$\eta_C^* = \frac{z_C \gamma_C \mu - \beta_C \gamma_C}{\beta_C} \tag{11}$$

which means as selection increases the compensated, plasmid containing bacteria will always dominate, as expected.

5 Ecologically competing compensation model

We briefly consider the model where we have 4 species competing ecologically. We note that there are a number of assumptions underpinning the validity of such a model, including that the plasmids cannot co-exist in a strain and that evolution has no role, and therefore the types are fixed by the initial conditions and the turnover dynamics.

This system has the dynamics

$$\begin{aligned}
\frac{df}{dt} =& \alpha f(1-f-c-p-q) - \mu f - \gamma_C fc - \gamma_P pf - \gamma_Q qf \\
\frac{dc}{dt} =& \beta_C c(1-f-c-p-q) - \mu c \\
\frac{dp}{dt} =& \beta_P p(1-f-c-p-q) - \mu p + \gamma_P pf + \gamma_C fc \\
\frac{dq}{dt} =& \beta_Q q(1-f-c-p-q) - \mu q + \gamma_Q qf,
\end{aligned} \tag{12}$$

which is a four ODE system. Sadly this set of differential equations does not admit any tractable analytic analysis, but we can use computational algebra to calculate fixed points and stability when we have known parameters, such as those presented in table 3 in the supplementary information. Of particular interest is the so-called (de)weaponisation case, when we set γ_C to a small value but leave all of the parameters as before. In this case the numerical competitions reveal a complex phase portrait for small values of γ_C, where the true fixed point is a damped oscillatory attractor in the interior which is competing with a weakly unstable fixed point of only compensated plasmids. The dynamics of the system are extremely slow and compounded by the presence of a second interior fixed point which is unstable and oscillatory. For this set of parameters determining of the system with a numerical only investigation would be extremely challenging.

References

1. Hall JPJ, Wright RCT, Harrison E, Muddiman KJ, Jamie Wood A, Paterson S, et al. Plasmid fitness costs are caused by specific genetic conflicts enabling resolution by compensatory mutation. PLoS Biol. 2021;19: e3001225.
2. Blazanin M. gcplyr: an R package for microbial growth curve data analysis. BMC Bioinformatics. 2024;25: 232.
3. Soetaert K, Petzoldt T, Setzer RW, Brown PN, Byrne GD, Hairer E, et al. Solvers for Initial Value Problems of Differential Equations ("ODE", "DAE", 'DDE') [R package deSolve version 1.40]. 2023. doi:10.32614/CRAN.package.deSolve
4. Soetaert K, Hindmarsh AC, Eisenstat SC, Moler C, Dongarra J, Saad Y. Nonlinear Root Finding, Equilibrium and Steady-State Analysis of Ordinary Differential Equations [R package rootSolve version 1.8.2.4]. 2023. doi:10.32614/CRAN.package.rootSolve
