# Supplementary material for: A chromosomal mutation is superior to a plasmid-encoded mutation for plasmid fitness cost compensation
Source: PLoS Biol. 2024 Dec 2;22(12):e3002926. doi: 10.1371/journal.pbio.3002926 (PMC11637435; doi:10.1371/journal.pbio.3002926)
Supplement: Attachment — Submitted filename: ResponseToReviewers_v6.docx [file pbio.3002926.s002.docx]

### Rebuttal to reviewers for manuscript PBIOLOGY-D-24-00147R1 Wright et al. Superiority of chromosomal compared to plasmid compensatory mutations

We thank all Reviewers and the Editors for their detailed and constructive feedback on our manuscript. Our revised manuscript incorporates various changes which address the Reviewer comments that we believe have greatly improved out work. Point-by-point responses to each Reviewer comment are provided below.

### Reviewer #1

In this study, Rosanna C.T. Wright and collaborators compare the ecological effects of chromosomal-encoded (chrCM) and plasmid-encoded (plaCM) compensatory mutations reducing the fitness costs associated with plasmids. The authors combine theoretical and experimental approaches to test the outcomes of the different CM in bacterial populations. They report that, in contrast to previous results in the field, chrCM are generally superior under a wide range of conditions. In general, I think that this is a very interesting paper and it combines modeling and experimental results in a very elegant way. The experimental system, which has been previously characterized by the authors, is now improved by using fluorescent tags and flow cytometry, which is a great addition. The results are novel, and uncover important ecological dynamics previously overlooked. The paper is beautifully written and the figures are clear and informative.

We thank the Reviewer for their supportive comments.

I have however a major concern:

The two main factors affecting the success of the different CM (plasmid or chromosome) are (i) the degree of compensation associated with the CM, and (ii) the rate of conjugation of the plasmid. The authors showed that chrCM alleviated the cost better than chrCM, but although the conjugation rate is the other key parameter, they proceed with the theoretical model and all the subsequent experiments without experimentally measuring it. Then, all the results suggest that one of the most important reasons for the plasmid carrying the CM not to increase in frequency in the population has to be that this mutation reduced the conjugation rate of the plasmid. Up to that point all the results make complete sense (including very elegant and robust experiments). But at the very end of the results section, the authors measure the conjugation rate of each version of the plasmid, and discover that they are basically identical (while the model predicts a big difference according to the
experimental results:  9.9 x 10-12 vs 1.3 x 10-13 ml.cells-1h-1). The authors suggest that "yet-to-be determined processes following plasmid establishment in recipient cells" should probably be responsible for the results, given that there is no difference in conjugation rate, and they are probably right, but they do not test them. The processes suggested by the authors in the discussion are: (i) a higher acquisition costs of plaCM compared to the wt plasmid or (ii) differences in the duration for conjugation derepression. Measuring acquisition costs is something that can be done relatively easily (as in Allison Lopatkin papers) maybe measure those? The relative relevance of secondary conjugations is more difficult to determine, but could also be done (by using recipient bacteria unbale to further propagate the plasmid, we do that using CRISPRi) or by performing mating assays with different frequencies of primary donors that may potentiate the effects of secondary
conjugations (but I see how this can be a lot of work and maybe beyond the scope of the paper). I think that the work would be much more robust if the authors try to find the reason why the compensated plasmid is less able to spread.

Like the reviewer we were also eager to unpick the reason why the compensated plasmid was unexpectedly ineffective in competition with the wild-type, and this comment motivated us to undertake some further analysis and experiments to investigate this further. We performed some exploratory analyses of models that included a delay class to investigate whether transient effects in newly-formed transconjugants could cause the cyclical rock-paper-scissors dynamic observed in the experiments. Although these models could not be solved analytically, we found that generally, as the presence of a delay class converts monotonic stable fixed points into oscillatory stable ones, this has the potential to expand the region of parameter space supporting cyclical dynamics in this system. We also performed further experiments. We prepared fresh transconjugants using our short (4 hour) conjugation protocol and immediately used these in growth curves, to test acquisition costs. As recommended by the Reviewer we also performed longer (18 hour) mating assays with a 100x excess of recipients to favour secondary conjugations. However, these efforts unfortunately did not solve the puzzle. We found no difference in conjugation rates between wild-type and plaCM plasmids, and though we detected a difference in growth rate of newly-formed transconjugants (acquisition cost), this favoured plaCM, in contrast to expectations. It is evident that considerable further experimentation would be required to resolve this unexpected result, likely necessitating a more in-depth understanding of the molecular mechanisms underlying plaCM, which is unfortunately beyond the scope of the current work. Our additional experimental results have been included in the revised manuscript, lines 443ff.

I guess that what I found a little disappointing as a reader, is that the entire paper builds around the results being explained by the difference in conjugation rate, which is finally not the case. Maybe a different structure (presenting experimentally determined conjugation rates at the beginning of the paper?) could change that feeling. In fact, the paper would still be very strong, because there are other important results and ecological dynamics described here.

We have revised the structure of the manuscript in line with the Reviewer’s suggestion and agree that this improves the flow of the paper, as well as accommodating the additional modelling and experimental results described above into the overall narrative.

Minor comments 

Figure 1. "a" and "b" are missing in the panels.

This has been fixed, thanks.

Figure 3 and 4 could be 2 panels of the same figure, and figure 5, 6 and 7 could also be panels of the same figure, in case there is a limited number of figures.

This would be easy to do, and we are happy to produce this figure if advised by the Editor, though we feel that the separate figures are currently easier to follow.

Figure 5: maybe indicate in the legend that there is no Hg selection.

Thanks, we’ve added this information for clarity.

Figure S6 Are the conjugation done under the same experimental conditions as the transfer experiments? In the methods it seems as if they could be a bit different (maybe this affects the rates?)

Good point — there are some differences between the transfer experiments and the conjugation experiments, namely, the use of glass vials in the transfer experiments vs. 96-well plates for the conjugation assays. This was necessitated by the requirement to track optical densities of many cultures during the course of the conjugation assays so as to ensure the experiments met the assumptions of the conjugation rate calculation. The use of different vessels has not obviously affected the measured rates as the calculated conjugation rates are very similar to those previously calculated from cultures grown over a longer period in glass vials (Figure S2). Further, for this to impact our conclusions, the vessel material would have to have a large affect on rates in a strain-specific manner, which seems implausible. This is now discussed line 716ff.

### Reviewer #2

In this manuscript, the authors perform both models and experiments comparing plasmid- and host-encoded mutations compensating for plasmid cost in P. fluorescens SBW25 and conjugative plasmid pQBR57. 
Contrary to model expectations, the experiments performed show that chromosomal CMs fare better than plasmid CMs, even in the presence of plasmid-free recipients. This is due to chromosomal CMs having reduced costs compared to plasmid ones, in competition in the absence of recipients, but more surprisingly also in the context of conjugation. Despite this, plasmid CMs do not have reduced conjugation rate compared to wt plasmids, as measured by classical assays. 

My main comment is that the conclusions (especially the title) should be nuanced:  the conclusions of the modelling are that a wider range of conditions can promote plasmid CMs vs chromosomal ones. This is tested experimentally with only one couple of mutations, and specifically only one plasmid CM. The authors find a higher benefit of the chromosomal CM to vertical transmission; and an unexpected cost of the plasmid CM to horizontal transmission. That is fascinating and investigated in detail here, but I don't think this unique example can lead to a plural in the title of the article - the generality of the findings depends on different plasmid CMs having the same consequences on transmission, this is not obvious and not shown here.

Thank you for the positive comments on our work. We have removed the plurals from the title as suggested.

It might also be worth discussing more how these results can explain observed experimental evolution results: in the previous paper(s) with this model system, were the chromosomal CMs obtained more frequently than the plasmid ones, in which conditions, did they invade faster?

Good idea — we don’t have invasion rate data, but we did observe that plaCM had a higher relative frequency in conditions where conjugation drove dynamics, and we have added some words to this effect in the introduction (lines 79ff).

Minor comments: 

L61 CMs "affecting" distinct genetic targets is confusing, as they can have (fitness) effects on both - replace with 'localised on' or similar to make it clearer?

Yes, good point. We have revised this (line 60ff).

L91-92 and later: what the authors mean could be made more explicit to place the cited papers in the context of CMs evolution - these papers do not directly deal with CMs 

We have rephrased the sentences around these citations to better explain the work reported in these papers (line 94ff).

Did the authors perform any measure of fluorescent marker cost by competing isogenic strains differing only in the markers?

Yes, we performed head-to-head competitions between SBW25::tdTomato(pQBR57) and SBW25::YFP(pQBR57). These resulted in fitness measurements very close to (and statistically indistinguishable from) zero (-0.03). Nevertheless, we corrected relative fitness estimates by this value to ensure comparability when different combinations of reference and test were used. This is now described in the methods (line 641ff), and with a supplementary figure showing the results of these and similar control competitions (Fig. S11).

Could the authors also discuss the amplitude of fitness effects compared to previous results? Selection coefficients ~ 1 in the absence of selection seem quite large. I had a rapid look at the previous PLoS Biology paper (Fig 2) where the amplitude seems lower but can't easily compare relative fitness W and selection coefficient results.

The current work includes a number of new comparisons that were not included in the previous paper, making direct comparison difficult. Nevertheless, some comparisons can be made. Plotting W instead of r, and swapping the test and reference strains in the plot, results in fitness values of SBW25(pQBR57) relative to SBW25::chrCM(pQBR57) of around 0.8, closely matching the previous measurement of the fitness cost of the uncompensated plasmid. See Figure below, in which panels describe the different reference strains, and x-axes the test strains. Given the differences with our previous work, however, we chose to use a different measure in the manuscript, but provide all raw data with both W and r calculated in the GitHub repository associated with the manuscript.

Not strictly required for the conclusions here, but if strains with the right markers are available, it would be interesting to see an experiment similar to Fig 6  where pQBR57 wt within the uncompensated ancestor competes with the wt plasmid in chrCM - that would show nicely if higher fitness in the donor leads to higher transmission overall.

This would certainly be an interesting experiment, but as the Reviewer acknowledges, is beyond the scope of the current manuscript.

L535 as the authors showed, the pleiotropic effects are not strictly on conjugation rate itself

Yes, good point — this has now been revised (line 534).

“the extent to which plaCM imposes pleiotropic effects on *plasmid transmission*”

Finally, maybe the two cited papers on acquisition cost could be discussed in a bit more detail. They do include modelling showing the effects of acquisition cost on conjugation dynamics, and showing that an intermediate acquisition cost can benefit a plasmid more than a low acquisition cost, which might be relevant to discuss here (I'm not sure how conceptually equivalent this is, but that appears at least similar to the 'paradox' of plasmid CMs not faring very well). 

We have reorganised this section, but have added a few more words discussing the conclusions of Ahmed et al. (2023) (line 543ff). These are very interesting studies which may well prove to be relevant to our system, but critically the results depend on transconjugant-lineage-level measurements of lag time and growth rate, features that we are currently unable to explore with our experimental system. We feel that prolonged discussion on the potential role that transconjugant-level acquisition costs might play in our system would be overly-speculative at this stage.

Reviewer #3

This paper combines modelling and experiments to investigate the relative success of chromosomal and plasmid-borne compensatory mutations for fitness costs of plasmid carriage. In general, there are a lot of things to like in this paper. In the modelling section, I found the general insights on the different roles of plasmid transfer for chrCM vs plaCM enlightening (i.e. reducing the fitness of your competitors through "weaponisation" vs direct spread of the CM). In the experimental work, especially the experiments showing RPS-like dynamics between plasmid-free, non-compensated, and plaCM cells and their direct link to model results stood out, as well as the results on the superiority of chrCM over plaCM in this system. These results have the potential to be insightful and relevant to a broad readership. However, I do have several important reservations towards the current manuscript that I think should be addressed.

Thank you for the supportive comments about our work.

One of the main results presented in the modelling section is that plaCM should succeed in its competition with plasmid-free cells and non-compensated carriers for a broader range of parameters than chrCM. I find the focus on this conclusion unfortunate for two reasons. First, as illustrated in Figure 1, this "broader range of parameters" is considered in terms of the (difference between the) conjugation rates of plasmids from uncompensated cells and from cells that have a CM. While for plaCM I can well imagine that there is a trade-off between fitness effect and transmissibility (as also found by Bethke et al, Dimitriu et al, and Turner et al; referred to on lines 168-169), for chrCM this is less obvious to me. In general, the fact that plaCM is present in the equilibrium for a broader range of the parameters considered does not say anything about the biological likelihood of these parameter conditions occurring (and hence of expecting to find these results). I would
therefore ask the authors to comment on the biological relevance of the parameter regime considered (especially the broad range of gamma_c, and the comparable range of gamma_c and gamma_q), and to reconsider whether their conclusion on the expected success of plaCM vs chrCM is justified. 

Regarding the Reviewer’s first point on the effect of chrCM, while the trade-off between horizontal and vertical transmission has been most directly described for plasmid-encoded genes (as indicated in the papers cited), there is also indirect evidence that chromosomal compensatory mutations could also affect conjugation rates. Conjugation rates vary with chromosomal genotype over many orders of magnitude even for the same plasmid, and there are clear examples of chromosomally-encoded alleles regulating plasmid transmission. Therefore including chrCM in these analyses is appropriate, not just for mathematical completeness, but also biological relevance. We now include a brief rationale for exploring chrCM effects on conjugation rate in the text (line 209ff).On the second point as to whether the broader range of parameters supporting plaCM are relevant, we are looking at the areas of Figure 1 that differ between plaCM and chrCM, supporting the presence of q but not c. This is principally the blue region of Fig 1B, which supports the plaCM, but the corresponding region of Fig 1A mainly does not support the chrCM. These are cases where conjugation rates of compensated and uncompensated plasmids are sufficient to allow invasion, but not dominance, and there is a trade-off such that the conjugation rate of compensated plasmids is reduced relative to the wild-type. This is now indicated in the text. The fact that many plasmids are maintained at intermediate frequencies in experiments (e.g. Lopatkin et al. 2017, Lundquist et al. 1986, as well as our previous work) suggests that this region of parameter space is biologically plausible, now indicated on line 179ff.

We now include plots showing the dynamics resulting from different regions of the illustrated parameter space. With plausible changes in conjugation rate (less than 2 orders of magnitude) we were able to explore all regions of the parameter phase diagrams with our experimentally-measured parameters. These are now shown in Figure S1.

We agree that it is important to ground model findings in biological reality, hence our efforts to establish an experimental system to test our model results.

Second, the focus on broad parameter ranges in my view hinders the comparison of model predictions and experimental data. All experiments are done on the same plasmid and CMs. Apart from the selection experiment, which does entail a change in eta, the experimental results hence basically hold for only one set of parameters (which the authors have actually measured!). The different experiments entail different initial conditions, but not changes in the parameters. Hence statements such as this one in the abstract "while plaCM was predicted to succeed under a broader range of parameters in mathematical models, experimentally chrCM dominated under all conditions" do not make sense to me. I think that throughout the manuscript, it should be made clearer what was varied in the different experiments (i.e. mostly who was initially present and in what fractions, and who was labeled) and how this relates to changes in the model (i.e. mostly changes in initial conditions, not
parameter values). This may also include reconsidering what the main message of the modelling section should be, such that it connects best to the experimental results.

The Reviewer is correct that all our experimental work is performed with a specific plasmid-host system, but we consider this a feature of our work, given that ours is a rare case where we can directly compare chrCM and plaCM in the same host-plasmid background. Most other reported plasmid-host pairings have yielded only chromosomal or plasmid CMs, making it hard to disentangle the effects of the specific host or plasmid from the effects of the CM. We would also highlight that different initial conditions do play a role in our modelling results (specifically the pink section in Fig 1A), and thus varying these in our experiments is a valid test of some of our modelling results. Nevertheless, we agree that our description of our findings could be improved here. We have modified the phrase in the abstract accordingly (line 31) and included more description of the experimental setup in the body of the manuscript (e.g. lines 374, 403).

A stronger connection between modelling section & experiments can also be obtained by making the modelling section more accessible to all readers. The way it is written now, I think the results in the modelling section will be very hard to understand for the broad readership of PLOS Biology. Specifically, I think more care can (and should) be taken to avoid and/or better explain terminology such as "the expect stable fixed point of the underlying system" (line 139) and "a linear addition to the stability conditions" (line 153). Also, I personally always find it very helpful if some example dynamics are shown, because such plots are much easier to interpret than plots like Figure 1. Especially for the RPS-like dynamics (e.g. like shown in Fig 8), this may help readers understand the dynamics of the model.

Thanks for pointing these out, we have simplified and/or removed the highlighted phrases, and agree that it improves the readability of the manuscript.

As mentioned above, we have also included a new Figure (new Fig. S1) that shows some example dynamics, and as our code is available and hosted as a Shiny app, it is easy to explore other elements of the dynamics. We have updated the link to the Shiny app so it points to a personal website (which currently redirects to the shinyapps.io website), providing robustness against server changes.

On the other hand, as a more mathematically inclined reader I was confused by some of the terminology used and I think this confusion can be avoided.

In several places, the authors state that the 4-equation system cannot be solved analytically, while the 3-equation systems can. To me, and I would think to many mathematically trained readers, solving a system of ODEs analytically typically means that analytical expressions are found for f(t), p(t), c(t), etc. This is however not what is meant: the equilibria of the system of ODEs and their stability are determined analytically.

This was in reference to a dynamical systems style approach where an analytic solution means a complete understanding of the ultimate fate of any trajectory, for any parameter set from any initial conditions. Complete analytic expressions for the time dependent trajectories are very rare outside of linearisable systems (e.g. as found in some electrical engineering approaches) or where there are exploitable symmetries (e.g. as found in some physical systems). A closed form expression for the fixed points is needed so that the Jacobian matrices in the linearisation proximal to the fixed points can be computed and decomposed to find the eigenvalues. This is always possible in 2D, sometimes in 3D and 4D but the algebra can become overwhelming, and almost never beyond that due to impossibility of solving a general quintic.

I was similarly confused by the use of the term "phase portrait" for Figure 1, because in a phase portrait I would expect to see state variables on the axes (e.g. "p", "c"), not parameters.

Apologies this was a slip due to the unfortunate naming convention we have. The referee is correct that phase portrait should refer to a state variable vs. state variable plot. A bifurcation plot is then a state variable vs. parameter plot and finally a phase diagram is a parameter vs. parameter plot, as was intended and now corrected here.

Lastly, I think more care should be taken in describing the results for the regime in which there is coexistence between f, p and q. It is not clear to me what the authors mean by a "linearised adaptive dynamics approach" (line 177 & SI), or why an AD approach could not pick up their result. If I understand correctly, in this parameter regime the "boundary" equilibria ((f, 0, 0), (0,p,0) and (0,0,q)) are unstable, right? Then I would think pairwise invasion analyses like the ones common in AD should pick up on the coexistence?

This is a complicated issue and we apologise for over simplifying. An AD approach, due to the multiple linearisations, indicates whether or not an invader is likely to grow (i.e. invade) or decline at the stable equilibrium of the resident. What it does not tell you is what the eventual fate of the system will be; there is an implicit assumption in AD that a successful invader will sweep to fixation if successful unless there are additional features in the PIP such as CSS or other saddle features where trajectories can diverge. Here by modelling the entire non-linear system until equilibrium we find the potential for a form of coexistence. The AD simply gives no information on the ultimate fate: whilst we may rule out unstable simple (or ‘boundary’) states the analysis does not describe any further details about the final state.

Also, both in the main text and the SI it is claimed that this coexistence occurs in a stable fixed point (spiral). However, this could not be shown analytically. How can you be sure the coexistence does not occur on some other attractor, like a limit cycle?

The Jacobian matrix for the fixed point can be analysed to determine this. Although the eigenvalues cannot be computed directly in principle the Routh-Hurwitz conditions can be used to determine that the characteristic polynomial of the relevant Jacobian always has negative roots. This means that there can be no unstable spiral (or focus) which is a necessary condition for the existence of a limit cycle (alternatively, this means that no Hopf bifurcation can occur to generate a limit cycle. The proof of this is long and not enormously illuminating but it can be shown the four relevant inequalities are all satisfied for the coexistence region where the (f*,p*,q*) fixed point is physical. A mathematica worksheet outlining this is now included.

In the model, spontaneous loss of the plasmid was not included, while this is sometimes done in these types of models. I do not think it is necessary to redo all the analyses including plasmid loss in the model, but I do think this assumption should be acknowledged and if possible, defended. Are spontaneous loss rates low for the specific plasmids considered? And if not, how would including spontaneous plasmid loss influence the results?

We considered this with some care. The addition of these terms greatly complicates the analytical progress but we have demonstrated in previous studies (Harrison et al. 2015) that we are still able to include it and make progress, but only in the simplest case. The experimental work showed that these events are rare on the timescales of this study and that population change is dominated principally by competition with existing plasmid-free cells (rather than their de novo generation by segregation). This rationale is included in the manuscript (line 137).

The experiments clearly show that for the specific system considered, chrCM is superior to plaCM. However, I am not fully convinced that the authors' claim that this is (partly) due to the weaponising of the plasmid (as is e.g. mentioned in the abstract) is justified. While the authors clearly show that this weaponisation can be important in the competition of chrCM vs plasmid free cells, in their particular system this effect may not play a major role in explaining the observed superiority of chrCM over plaCM because plaCM has low fitness anyway, and cannot fully spread in a susceptible population. Is the superiority of chrCM over plaCM then not just due to the difference in fitness (disregarding the weaponising effect)? I think the model offers a great opportunity to test this, namely by simulating the competition between plaCM and chrCM for gamma_c = 0. If chrCM cannot take over under those conditions, the conclusion can be drawn that the weaponising effect is necessary
to explain the observed results.

We conducted the suggested analysis in more detail. The key parameter is the growth rate of the two competing mutated strains and if this is equal the mathematics leads to various non-hyperbolic fixed points for which the linear analysis does not apply. If the fitness of the two is the same then initial conditions determine the outcome due to the independence between the plaCM and chrCM strains. If the growth rate of the chromosomal mutant is less than the plasmid borne one then simply the chromosomal mutant cannot invade. An interesting feature is if we allow the chromosomal mutant to have a superior growth rate (but no conjugation), then a RPS cyclical dynamic occurs, now discussed in line 219ff. The general case for gamma_c=0 is essentially the x axis of Fig 1A and as can be seen the chromosomal mutant can never take over entirely but can be part of a mixed solution when gamma_p is high, due to the likelihood of it having a higher growth rate than the uncompensated plasmid containing strain which would otherwise dominate.

One of the final conclusions of the paper is that, because chrCM is superior to plaCM, one may expect to find "hubs" of plasmid carriers because their chrCM allows for many plasmids. However, while in the system studied in this paper chrCM was indeed superior to plaCM, we are left wondering how general this result is and it would be good if the authors could critically discuss this. Again, the model may provide additional opportunity here: under what parameter conditions do you expect chrCM to be superior to plaCM? And should we expect these parameter conditions to be often met in nature?

Thank you for raising this important point. Indeed, our model was developed exactly as a means of generalising beyond our specific study system, and we describe the parameter conditions under which chrCM and plaCM might invade and dominate a population (sections beginning line 143 and 169 respectively). We also provide a numerical model for readers to interactively explore different parameter sets (Figure S8 and SI Text line 125ff). One of our key conclusions is that while plaCM can in principle succeed under a broader range of parameters, in reality this advantage was not seen as evidenced by the head-to-head competition experiments we performed (particularly Fig. 7). As to whether these conditions are often found in nature, this is a more difficult question to evaluate directly precisely because of the lack of experimental data measuring and comparing the effects of both plaCM and chrCM for different plasmid-chromosome pairs. Which CM mechanisms predominates will ultimately come down to the extent to which each CM ameliorates fitness costs and whether it trades off against transmissibility, and we present several arguments for why chrCMs are likely to be more effective in this regard in the discussion (e.g. lines 487ff, 503ff, 553ff). Though some studies have managed to unpick the molecular bases of plasmid fitness costs and resolution across different bacterial-plasmid-CM pairings, further work is clearly required in this area. We have made some amendments to the discussion to clarify our arguments here (e.g. section beginning line 531) and we also removed the plural from the title to clarify that we are experimentally testing only a single pair of CMs.

I would like to end by stressing that I am not raising all these points to burn down the paper, but rather because the paper interested me and I would like to see the information in it become available for a broad audience. I hope these comments can contribute to doing that.

Thank you for your thoughtful comments — we believe the changes we’ve made to the manuscript in response have indeed improved it for such an audience.

MINOR TEXTUAL COMMENTS / TYPOS

Lines 52-58 & 60-62 basically state the same thing twice -> consider reducing the text here?

Thanks, we have edited this section.

Line 95: "3-equation ODEs" -> remove first "equation"?

Again, thanks for the suggestion. We’ve made the edit.

Line 151: beta_q is mentioned here but this has not yet been defined.

Thanks for picking up on this, this is a typo and has been corrected.

Line 185, end: "though" -> "through"

Well spotted — thanks.

Line 283: "previous worked" -> "work"

Corrected, thanks

SI, Basic model: "grown rate" -> "growth rate"
SI, Plasmid compensation model, page 3: "This fixed point exists in the band ..." -> "This fixed point is positive in the band ..." (at least, my strong suspicion is that this fixed point also exists for other parameter values, just not in the positive quadrant.)

Both corrected, thankyou
