# Supplementary material for: A chromosomal mutation is superior to a plasmid-encoded mutation for plasmid fitness cost compensation
Source: PLoS Biol. 2024 Dec 2;22(12):e3002926. doi: 10.1371/journal.pbio.3002926 (PMC11637435; doi:10.1371/journal.pbio.3002926)
Supplement: Attachment — Submitted filename: Revision2_Response_v4.docx [file pbio.3002926.s003.docx]

### Response to reviewers for manuscript PBIOLOGY-D-24-00147R2 Wright et al. Superiority of a chromosomal compared to a plasmid compensatory mutation

Thank you to the Editor and all Reviewers for helpful and constructive comments on our manuscript. We believe it is greatly improved following the changes we have made in response to the suggestions.

### Editor

a) Please make your Title more explicit by changing it to "Chromosomal mutations compensate for the costs of carrying a plasmid better than plasmid-encoded ones."

We have carefully considered the alternative title, and would like to note that, while it may be more explicit than the current one, it also introduces some new issues. Namely (a) it suggests that the focus of the work is about the extent to which mutations reduce fitness costs, whereas the principal object is more general (i.e. to understand the success of the different compensatory mutations, which includes their effects on transmission under varying conditions as well as fitness costs), and (b) it re-introduces the issue of generality that was raised by a previous review. We would therefore suggest an alternative: “Superiority of a chromosomal compared to a plasmid-encoded mutation for plasmid fitness cost compensation, owing to higher efficiency amelioration and weaponisation of costly plasmids through conjugation into competitors”, but if the Editor’s suggestion remains the preferred option we will change it.

b) Please attend to the remaining concerns from the reviewers. Reviewer #1 now checks Accept and has no further concerns. Reviewer #2 simply has one textual request. Reviewer #3, however, has a few remaining points; one is a presentational query about Fig 1, and the other relates to a problem that s/he encountered when running your model (and might require further analysis).

Please see our response to each Reviewer’s point in turn below.

c) Please address my Data Policy requests below; specifically, we need you to supply the numerical values underlying Figs 2, 3, 4, 5, 6, 7, S1ABC, S2, S3, S4, S5, S6, S7, S8, S9, S10, S11, either as a supplementary data file or as a permanent DOI’d deposition. I note that you already have data and/or code in depositions in Github, Liverpool DataCat and CEH, as well as in the zipped supplementary folder. There seems to be quite a lot of overlap between these, so it might be best to focus on one of them as place to direct readers for the data and code needed to recreate the Figures. It seems that Github might be the most comprehensive, as it contains the code and the Shiny app? However, because Github depositions can be readily changed or deleted, please make a permanent DOI’d copy (e.g. in Zenodo) and provide this URL (see below).

The flow cytometry data is archived at CEH to comply with funding policy, and the deposition at the University of Liverpool is likewise institutional policy. However, we have prepared a permanent DOI’d copy in Zenodo following your suggestions (thank you for this) and use this throughout: <https://dx.doi.org/10.5281/zenodo.13963497>

d) Please cite the location of the data clearly in all relevant main and supplementary Figure legends, e.g. “The data underlying this Figure can be found in S1 Data” or “The data underlying this Figure can be found in <https://zenodo.org/records/XXXXXXXX>

This information has now been added for each Figure. The README.md file provided and the associated analysis scripts provided in the Zenodo deposition detail how to reach the individual data sheets (Zenodo does not allow direct linking to individual files in the archive, and so the hyperlink points to the record front page).

e) Please make any custom code available, either as a supplementary file or as part of your data (Zenodo?) deposition.

All analysis code used to reach conclusions from raw data is provided as part of the GitHub repo, now archived at Zenodo: <https://dx.doi.org/10.5281/zenodo.13963497>

### Reviewer #1

[identifies himself as Alvaro San Millan]

I am happy with the modifications made by the authors.
Alvaro San Millan


Reviewer #2

The authors have satisfactorily responded to my comments. In general I also thought the new version read better following the reshuffling based on other reviewers comments.  

I am still curious after the addition of lines 80-82 in the new version. If plaCM was the predominant CM in populations in which the plasmid persisted by infectious transfer - this suggests it was still more fit in the conditions of that given evolution experiment, despite this not being the case in the experiments presented here. Was there some difference in the design of both experiments that could suggest an explanation for this, which could be mentioned in the discussion? 

The Reviewer raises an interesting point, i.e. that given its limitations, why was plaCM observed at all in our evolution experiments? There are several possible explanations, but as they are all speculative and would require a prolonged re-iteration of the design and results of our evolution experiment we have decided not to include an extended discussion here. Our favoured hypothesis is that the accessibility of the plaCM may be enhanced by the action of other MGEs in our system, namely the potentially higher susceptibility of the AT-rich plasmid to acquire disruptive transposon insertions, and we include a couple of words on this in the discussion (line 568ff).

Reviewer #3

The authors have addressed most of my comments very well, and I thank them for their efforts. The model section is now easier to read, and in general the results are well-explained. I also enjoyed the shiny-app, which I now explored a bit more. However, there are still two points that I think should be addressed before publication.


Point 1 (minor): Grey area in figure 1

a. I forgot to comment on this during the first round (for which I apologise), but it its unclear to me why the grey areas in Fig 1 (where gamma_C/Q > gamma_P) do not start from the origin, but rather from higher on the y-axis? Is the intersection of the axes not at gamma_C/Q = gamma_P = 0? If so, this should be indicated along the axes or in the legend. If I misunderstood and the intersection of the axes is actually gamma_C/Q = gamma_P = 0, I would recommend adjusting the text in the legend describing the grey area to avoid confusion.

The graph shown is intended to be purely analytic and focusses on the region of interest. If the graph was shown numerically, it would be almost impossible to interpret, as is normally the case for numerically produced phase portraits, hence why there is no numerical references on it (it is actually plotted for x between 0.005 and 0.32, and y between 0.00 and 0.22, with mu = 0.04, betaP = 0.63, betaC = 0.75). The intersection is (0,0), but since this assumes no conjugation, we did not focus on this area. Instead, we focus on the region of interest shown as we know there are no further complications, such as bifurcations or similar outside of this region, and so for parameters falling outside of these bounds the answers can be safely extrapolated. We now explain this in the legend to Fig. 1 (line 235ff).

b. In the legend of Fig 1, it is stated that in this grey area "the wild-type plasmid is always lost with the system reverting to the well known two-member-model of plasmid-free and plasmid containing type, as described in the Suppl Appendix." If I understand correctly, though, this is not entirely correct. For the plaCM model, yes, but for the chrCM the basic model in the suppl appendix does not hold as conjugation with chrCM cells as donor does not produce more chrCM cells, but rather plasmid-carrying, non-compensated cells. This is also the reason why you get bistability for any value of gamma, as is explained in the manuscript (lines 157-167). This point is easily remedied either by removing the grey areas from the figures in Fig 1 and showing the phase diagram for the whole space (which would have my preference for completeness), or by altering the text in the legend explaining why the gamma_C/Q > gamma_P condition was considered irrelevant.

This is correct and well observed. In both cases a model can of course be created with gamma_Q > gamma_P or gamma_C > gamma_P. In the former case a relabelling symmetry exists which means this is simply not interesting due to the sign of Omega changing, which is described in the text (line 187ff in the revised text), and hence the reason for the grey area (if you choose a value in the grey area, it is equivalent to switching the wild-type and plaCM labels). But for the latter case the referee is correct as this structural symmetry is not present. This is an oversight in our part: we did not consider it to be biologically likely for a chromosomal mutation to decrease fitness costs and increase conjugation rate, but there is nothing in the mathematics that discounts this possibility, and on reflection there are some mechanisms that might lead to this result. We have therefore re-drawn this plot without the grey triangle, but the regions are otherwise unchanged, and included a brief explanation (line 223ff).

Point 2 (somewhat more major): Inability of the model to replicate experimental results

This point arose from some simulations I did based on a misunderstanding of the point I rose earlier about whether the "weaponisation"-effect actually plays a role in the experimental system under study. While the authors clearly explain what would happen in a system with only plaCM and chrCM present, and in the "chrCM-only" model when gamma_c is equal to zero, I actually meant to suggest to test this condition in the full, simulated system (i.e. with both plaCM and chrCM present, as well as plasmid-free cells and non-compensated plasmid-carriers - so a "f + p + c + q"-model mimicking the experimental conditions of e.g. Fig 3). My hypothesis was that if one could replicate the experimental results (i.e. the superiority of chrCM) while gamma_c was equal to zero, this would suggest that the weaponisation was not important in this specific case, while if one could not find chrCM-superiority for gamma_c = 0 but this could be found for gamma_c > 0, this would indicate that the weaponisation effect is necessary to explain the experimental results. When I did some simulations* to try and test this myself, however, I ran into the problem that I was unable to replicate the experimental results of e.g. Fig 3 and 5 with the model, even when plugging in much lower conjugation rates for plaCM (100 fold lower; as was e.g. done in the paper for Fig S8 to mimic the RPS-dynamics found in Fig 7; or even 1000-fold lower). Maybe I didn't try hard enough or made a mistake - if so I would be happy to be corrected. If not, however, I think this is a problem, because in the discussion it is suggested that the difference between experimental results and model predictions can likely be explained by a yet-to-be-unveiled mechanism that would lower the effective conjugation rate of plaCM, but the simulation exercise suggests that something else is going on. I recognise that it is beyond the scope of the current work to do a full analysis of the f-p-c-q-model, but I do
think that it should be acknowledged that as the model currently stands, it is not able to explain the observed superiority of chrCM even if plaCM would have much lower conjugation rate. I would therefore urge the authors to use their already existing simulation set-up to simulate the experiments of Fig 3 and Fig 5 with their measured parameters but much lower conjugation rate for plaCM (as they did in Fig S8 for the experimental results of Fig7) and then to comment in their discussion about any discrepancies between modelling predictions and the experimental results.

* What I did was simulate the f-p-c-q-model using the parameters in Table S1 (including the correction of conjugation rates for carrying capacities, as nicely explained in the table) and then simulate dynamics for different initial conditions (including ones with high values of c, to avoid missing c-dominated equilibria that are only reached above a certain manifold) to get a sense of the possible dynamics and equilibria. I could easily replicate the RPS-dynamics of Fig 7 and S8 by plugging in a 100-fold lower conjugation rate for q, but if I then took these parameters (with gamma_c = gamma_p) to simulate dynamics of the f-p-c-q-model I found that it still predicted fixation of q. When lowering q's conjugation rate even more (1000-fold), I found some large amplitude oscillations, but in all of the simulations I did this still ended with c disappearing from the system.

The reviewer raises several important points here. First, regarding the correspondence between model predictions and our experimental data, specifically whether the data shown in Figures 3, 5 and 6 are predicted with relevant parameters in our model. I re-ran the models using the parameters described in Table S1, with initial conditions resembling the experiments. First, for Figure 3 (I focus here on Figure 3a, but similar results are obtained by increasing selection):

**Figure R1. (A)** Excerpt from Figure 3 from the main paper showing the experimental results. **(B)** Results from numerical simulations using the model described in the Shiny App in which plaCM corresponds to CM1 and chrCM corresponds to CM2, and $\gamma_{Q}= \gamma_{C}$. **(C)** Results from numerical simulations in which $\gamma_{Q}= {0.01\gamma}_{C}$ (as discussed in the revised text line 448ff). Model results are cropped to a time of 1000, both models ultimately result in fixation of CM2 (chrCM). CM2 transconjugants are shown in grey to highlight similarities with the experimental data, which was incapable of distinguishing between chrCM transconjugants and plasmid-free recipients.

**//**

Fig. R1 shows that the modelling results indeed resemble the figure in several aspects, particularly if the effective conjugation rate of plaCM is reduced as discussed in the manuscript. Specifically, we can see a transient benefit of plasmid-free as the population initially expands, and the ultimate success of chrCM over plaCM and plasmid-free.

Next, for Figures 5 and 6 the same analysis was performed (since with the modelling it is possible to distinguish transconjugants and donors):

**Figure R2. (A)** Reproduction of Figure 5 from the main paper showing the experimental results. **(B)** Reproduction of Figure 6 from the main paper showing the experimental results. **(C)** Results from numerical simulations in which $\gamma_{Q}= {0.01\gamma}_{C}$ (as discussed in the text). The ultimate outcome of these simulations was fixation of chrCM. **(D)** Results from numerical simulations in which $\gamma_{C}=0$. Here, chrCM fixed only in the absence of plasmid-free, and was otherwise lost from the system.

*//*

*Again, Fig. R2C shows modelling results that resembles the experimental data (shown in Fig. R2A and R2B). Specifically, we can see the emergence of chrCM was preceded by an invasion of transconjugants from chrCM, particularly in the 10:1 plasmid-bearing:plasmid-free treatment, that reduced the relative fitness of the competitor relative to chrCM, enabling subsequent invasion. Overall, we consider there to be a good correspondence between the modelling and experimental results presented in the manuscript. These results can be obtained from our Shiny app with the following parameters.*

**Figure R3.** Panels from the Shiny App showing the results presented in Figure R2C. Note that the ordering of the subpopulations is different in this plot (Fig. R2C was manually adjusted to resemble the experimental plots).

//

Without seeing full details, the reason for the divergence between our results and those described by the Reviewer is unclear. We have put all these analyses on our GitHub repository (and checked these several times) so don’t believe it is an error at our end. The consistency between our simulations and the general analytical results also gives us confidence that our simulation models have been formulated correctly. However, if any issue can be identified we would be grateful and happy to address it.

Next, the Reviewer proposes an interesting analysis: to use the model to investigate the contribution of conjugation to the success of chrCM and test the suggested mechanism of plasmid ‘weaponisation’. We conducted this analysis by setting the conjugation rate from chrCM ($\gamma_{C}$) to zero in our model, and keeping all other parameters constant. The results are shown in Fig. R2D, which can be compared directly with R2C. These show that chrCM (and the plasmid overall) was not able to persist in competition with plasmid-free in the absence of conjugation, demonstrating the importance of weaponisation in the model, and thus its likely importance in our experiments. We thank the Reviewer for this suggestion, and we believe that including this result would improve the manuscript further, so we have added a brief explanation and Supplementary Figure (lines 396ff).

Though it is not possible to analytically solve the four-equation model, we can calculate fixed points for a specific set of parameters. Whilst not a general solution, as we do for the 3 model where we can construct an analytic phase diagram, it does enable us to determine the presence of unstable fixed points in the interior and the likely slow dynamics of approach to the final solution, both of which will contribute to complicated and potentially confounding temporal trajectories of purely numerical solutions. Using the experimentally-measured parameters, we can again show that extent of compensation is the prevailing factor determining the success of competing CMs, as indicated in the revised manuscript (lines 249ff) and Supplementary Appendix.
